# Peritoneal Tuberculosis: A Forsaken Yet Misleading Diagnosis

**DOI:** 10.1155/2019/5357049

**Published:** 2019-11-04

**Authors:** Joseph Kattan, Fady Gh. Haddad, Lina Menassa-Moussa, Carole Kesrouani, Stephanie Daccache, Fady G. Haddad, David Atallah

**Affiliations:** ^1^Hematology and Oncology Department, Faculty of Medicine, Saint Joseph University, Beirut, Lebanon; ^2^Radiology Department, Faculty of Medicine, Saint Joseph University, Beirut, Lebanon; ^3^Pathology Department, Faculty of Medicine, Saint Joseph University, Beirut, Lebanon; ^4^Internal Medicine Department, Faculty of Medicine, Saint Joseph University, Beirut, Lebanon; ^5^Obstetrics and Gynecology Department, Faculty of Medicine, Saint Joseph University, Beirut, Lebanon

## Abstract

In women presenting with an abdominal mass and ascites, the first diagnosis to consider is ovarian cancer. However, clinicians should always consider alternative differentials, namely, peritoneal tuberculosis, especially in the presence of respiratory symptoms and with the increasing prevalence of extrapulmonary tuberculosis. Peritoneal tuberculosis can mimic the clinical presentation of ovarian cancer, and on imaging, it can show similar features of peritoneal carcinomatosis and nodules. Tumor markers can also be elevated in the absence of malignancy. We present the case of a 44-year-old woman with abdominal distension and ascites. Imaging with CT scan, MRI, and PET scan were inconclusive, showing peritoneal nodules. Cytology of ascites was negative. Laparoscopy was done showing Koch bacilli followed by pulmonary sampling showing *Mycobacterium tuberculosis*. The patient was treated with quadritherapy with resolution of symptoms.

## 1. Case

We report the case of a 44-year-old multiparous woman, without previous medical history, having a positive family history for breast cancer in her paternal aunt. She presented elsewhere for a one-month history of cough and abdominal distention, followed by episodes of fever at 39°C. Abdominal ultrasound was done, showing large ascites and a right ovarian mass.

An abdominopelvic MRI was done showing a hyperintense, heterogeneous mass of the right ovary, measuring 20 × 17 mm, with restriction of diffusion, in favor of a malignant process.

Tumor markers showed an elevated CA 125 level of 543 and normal CA 19-9 level of 13.2. Serum C-reactive protein (CRP) was 53.

A diagnostic abdominal tap was done with cytology examination showing no malignant cells in the peritoneal fluid.

A PET/CT scan was also done revealing peritoneal thickening and diffuse peritoneal fixation with hypermetabolism at the right ovary (SUV = 6.7) of 2 cm, as well as bilateral pleural fluid that was not hypermetabolic.

**Figure 1:**
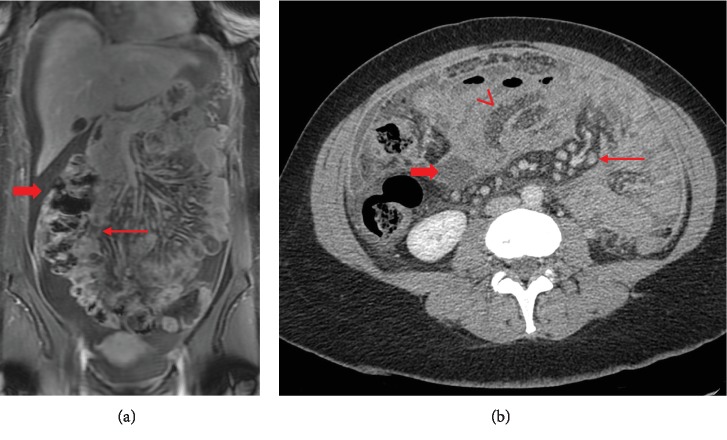
(a) Coronal fat sat T1-weighted MR image with intravenous contrast injection showing parietal thickening of the terminal ileum (long arrow) and abdominal ascites (thick arrow). (b) CT scan showing mesenteric adenopathy (long arrow), thickening of the ileum (arrowhead), and mesenteric fluid (thick arrow).

The patient was admitted at our department for further investigations. New work-up with total body CT scan was done showing moderate enhanced ascites associated with mesenteric fat streaking with millimetric nodules suggestive of peritoneal carcinomatosis (Figure 1).

Abdominal tap was repeated revealing only inflammatory reaction and serous fluid without evidence of malignant cells and with negative culture. An ultrasound-guided Tru-cut biopsy of the peritoneum was performed and revealed only inflammatory changes without malignant cells. Right pleural fluid was aspirated and sent for cytology and culture. Analysis showed no evidence of bacteria, infection, or malignant cells.

**Figure 2:**
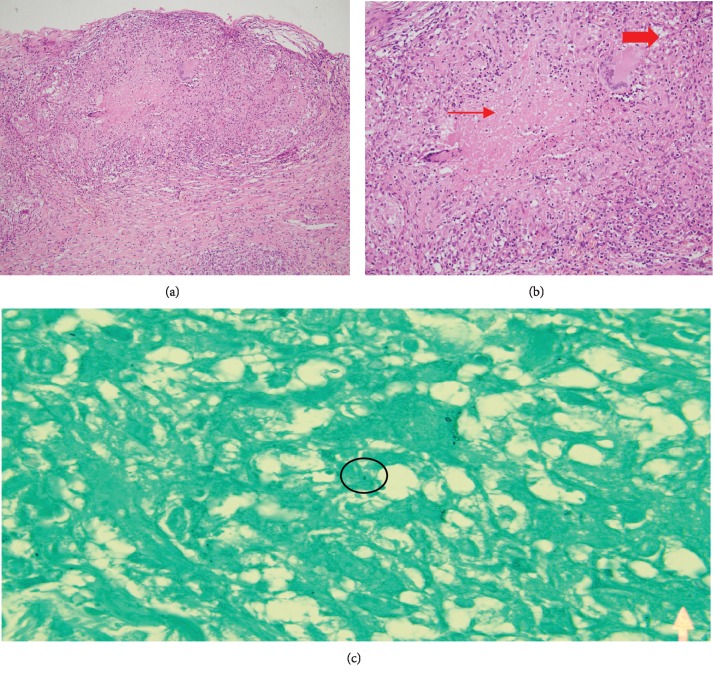
(a) Pathology specimen revealing granulomatous tissue; (b) presence of multinucleated giant cells (thick arrow) and areas of necrosis (long arrow); (c) Ziehl–Neelsen staining showing a comma-shaped acido-alcohol resistant bacillus compatible with Koch bacillus (black circle).

Ultimately, a laparoscopic exploration was done with multiple biopsies taken from peritoneal nodules. Histological result showed a necrotizing, epithelioid, and gigantocellular granulomatous reaction, with Ziehl staining showing exceptional acido-alcohol resistant bacilli compatible with Koch bacilli (Figure 2). Subsequently, pulmonary sampling was done and samples were analyzed for *Mycobacterium tuberculosis* by polymerase chain reaction (PCR), with a negative result showing no sign of pulmonary infection.

The patient was started on quadritherapy (rifampicin, isoniazid, ethambutol, and pyrazinamide) for 2 months with significant improvement in her clinical picture and weight gain of 4 kg and will continue for an additional 6 months of biotherapy with rifampicin and isoniazid. Follow-up imaging with abdominal MRI was done showing no ascites or masses in the abdomen.

## 2. Discussion

In women presenting with abdominal discomfort, abdomino-pelvic mass, weight loss, ascites, and elevated levels of CA 125, the first diagnosis to be afraid of is ovarian cancer. However, abdominal tuberculosis can present with the same vague symptoms, and it is an important differential diagnosis to consider and to hope in a minor subset of lucky women [1]. Worldwide, the prevalence of extrapulmonary tuberculosis is increasing parallel to the rise of acquired immunodeficiency syndrome (AIDS), mainly in developing countries, with around 12% of abdominal involvement [2]. Peritoneal tuberculosis is a rare entity in developed countries but should always be considered in developing countries, accounting for less than 1% of tuberculosis cases [3].

In our patient, nonspecific cough and fever could point towards an infectious process, namely, tuberculosis. However, chest X-ray, chest CT scan, and microbiological pulmonary cultures with PCR returned negative for *Mycobacterium tuberculosis*. In addition, the elevated CA 125 and the ovarian mass along with peritoneal infiltration in a middle-aged woman made the diagnosis of ovarian cancer more likely.

The elevated level of CA 125 to a value of 543 in our case was misleading and orienting towards an ovarian cancer. In fact, the median CA 125 level among ovarian cancer patients is around 400 [4]. This nonspecific marker may cause confusion, as it is elevated in a variety of conditions such as infections, tuberculosis, endometriosis, Meigs syndrome, menstruation, ovarian hyperstimulation, and a number of nongynecologic conditions like active hepatitis, acute pancreatitis, pericarditis, or pneumonia [5].

Abdominal imaging (CT scan and MRI) shows similar features for peritoneal tuberculosis and carcinomatosis, making differential diagnosis difficult. Both conditions present with micro- or macronodules in the mesentery, as well as omental and parietal peritoneal anomalies, thus suggesting a possible similar mechanism of disease spread via rupture of a mesenteric lymph node or through a ruptured capsule of an ovary, in the cases of tuberculosis and ovarian cancer, respectively [6]. Usually, adenopathies from ovarian cancer start in the retroperitoneum; there was no retroperitoneal adenopathies in our patient. Carcinomatosis is predominant in the peritoneum, not in the mesentery, and thickening of the bowel walls is usually irregular in ovarian cancer and rarely as regular and diffuse as in our case. Bowel thickening in abdominal tuberculosis is predominant in the terminal ileum and in the cecum [7].

PET/CT scan is being increasingly used in cancer staging and identifying malignant lesions. It was also shown to be useful for differentiating between tuberculous peritonitis and peritoneal carcinomatosis. Tuberculosis is more likely when the tracer distribution was uniform and smooth, with more than 4 regions involved and a string-of-beads 18F-FDG uptake, whereas peritoneal carcinomatosis was more prevalent when 18F-FDG uptake was clustered and focal, with irregular thickening and nodular regions [8]. Nevertheless, all these imaging modalities are not completely sensitive nor specific in this setting as shown with our patients that turned out to have peritoneal tuberculosis, after CT scan, MRI, and PET/CT scan pointed towards an ovarian cancer and associated peritoneal carcinomatosis.

Our patient was highly suspicious of ovarian cancer, but multiple abdominal taps returned negative for malignant cells. Despite the low negative predictive value of ascites in diagnosing ovarian cancer, repetitive negative cytology in the presence of an ovarian mass with peritoneal thickening and a febrile episode should guide the clinician to search for a tuberculosis infection, since both cases present with an exudative liquid. Hence, our patient could have benefited from ascitic fluid analysis by PCR for a mycobacterium complex which is a reliable method for diagnosis. Moreover, ascitic fluid adenosine deaminase activity (ADA) has a sensitivity of 100% and specificity of 92-100% in the diagnosis of peritoneal tuberculosis [9], thus obviating the need for more complex procedures.

In spite of thorough investigations, exploratory laparoscopy and/or laparotomy may be necessary for ruling out ovarian malignancy or confirming abdominal tuberculosis [1]. As in our case, despite repetitive nonconclusive and negative investigations, surgical biopsy was finally diagnostic of a tuberculous granuloma.

After the diagnosis of peritoneal tuberculosis, patients treated with antitubercular therapy should be monitored for treatment response, either clinically or biologically. An important parameter is the CRP level which is elevated early in the course of the disease and declines progressively during therapy. Failure of CRP decline indicates drug-resistant tuberculosis or alternative diagnosis such as peritoneal carcinomatosis and inflammatory bowel disease [10]. In our patient, serial CRP measurements should have been done in order to evaluate response to treatment.

## 3. Conclusion

This case highlights the importance of raising the possibility of alternative, although rare, differential diagnoses when evaluating women with ovarian cancers. Peritoneal tuberculosis should be considered in women presenting with ascites, radiographic images of peritoneal nodules, and elevated CA 125 levels, even if the clinical picture is suggestive of malignancy in a reproductive woman. Clinicians should combine blood tests, abdominal imaging, and microbiological tests in order to make the correct diagnosis. However, the tissue biopsy remains the gold standard for diagnosing peritoneal tuberculosis when all other tests are negative or inconclusive, thus allowing appropriate treatment with antibiotics.
